# A dual isotopic approach using radioactive phosphorus and the isotopic composition of oxygen associated to phosphorus to understand plant reaction to a change in P nutrition

**DOI:** 10.1186/s13007-017-0227-x

**Published:** 2017-09-25

**Authors:** Verena Pfahler, Federica Tamburini, Stefano M. Bernasconi, Emmanuel Frossard

**Affiliations:** 1 Department of Environmental Systems Science, ETH Zurich, Eschikon 33, 8315 Lindau, Switzerland; 2 Sustainable Agriculture Sciences, Rothamsted Research, Okehampton, Devon EX20 2SB UK; 3 Department of Earth Sciences, ETH Zurich, Sonneggstrasse 5, 8001 Zurich, Switzerland

**Keywords:** δ^18^O_P_ of TCA P, ^33^P, Phosphorus, Radioisotopes, Soybeans, Stable isotopes

## Abstract

**Background:**

Changing the phosphorus (P) nutrition leads to changes in plant metabolism. The aim of this study was to investigate how these changes are reflected in the distribution of ^33^P and the isotopic composition of oxygen associated to P (δ^18^O_P_) in different plant parts of soybean (*Glycine max* cv. Toliman). Two P pools were extracted sequentially with 0.3 M trichloroacetic acid (TCA P) and 10 M nitric acid (HNO_3_; residual P).

**Results:**

The δ^18^O_P_ of TCA P in the old leaves of the − P plants (23.8‰) significantly decreased compared to the + P plants (27.4‰). The ^33^P data point to an enhanced mobilisation of P from residual P in the old leaves of the − P plants compared to the + P plants.

**Conclusions:**

Omitting P for 10 days lead to a translocation of P from source to sink organs in soybeans. This was accompanied by a significant lowering of the δ^18^O_P_ of TCA P in the source organs due to the enzymatic hydrolysis of organic P. Combining ^33^P and δ^18^O_P_ can provide useful insights in plant responses to P omission at an early stage.

## Background

Phosphorus (P) is an essential nutrient for plants. It is taken up by the roots as phosphate and is translocated to the leaves and other plant parts through the xylem and phloem [1, 2]. In plants with a high P status, a large proportion (85–95%) of phosphate in cells is stored in the vacuole (non-metabolic P), whereas only 5–15% are considered as metabolic P, i.e. P taking part in plant metabolism [3, 4]. When P supply becomes limiting for plant growth, P stored in the vacuole is mobilised and translocated from old leaves (source) to growing plant tissues (sinks) like developing leaves and fruits [5, 6]. Plant responses to a low P nutrition status also include replacement of phospholipids by sulfo- or galactolipids, the activation of acid phosphatase, a higher expression of high affinity P transporters, and a decrease in nucleic acid concentration [7–11].

The use of ^32^P and/or ^33^P can elucidate P translocation from developed to developing leaves in plants [1, 3, 12, 13]. Before P is translocated it needs to be mobilised, either from P stored in the vacuole or via enzymatic hydrolysis of organic P compounds. The stable isotope composition of oxygen associated to phosphorus (noted thereafter δ^18^O_P_) is altered by enzymatic processes and thus could provide information on these processes. Enzymes can exchange one to four oxygen (O) atoms bound to P with O of ambient water [14–16]. The inorganic pyrophosphatase (inorganic PPase), an ubiquitous enzyme regulating the pyrophosphate level in cells, is responsible for a temperature dependent equilibrium between O in water and phosphate [17–19]. Also, other acid anhydride hydrolases involved in the transport of nutrients across membranes, could lead to equilibrium between O in water and in phosphate [20]. Enzymes, which catalyse the hydrolysis of organic P compounds such as acid or alkaline phosphatase, release phosphate with a different δ^18^O_P_ compared to the original organic P compound due to isotopic fractionation [21, 22]. Alkaline and acid phosphatases, 5′-nucleotidase, and deoxyribonuclease 1 preferentially incorporate ^16^O from water into phosphate, resulting in the release of phosphate depleted in ^18^O compared to the original organic P compound [16, 21, 22].

We previously investigated the ^18^O enrichment of trichloroacetic acid soluble reactive P (TCA P; mainly phosphate [23]) extracted from soybean leaves (*Glycine max* cv. Toliman) grown under P sufficient conditions in the glasshouse. We found that the δ^18^O of leaf water was the main control of δ^18^O_P_ of TCA P in soybean leaves under our experimental set-up [23].

We assess here the effects of stopping the P supply in the nutrient solution on the δ^18^O_P_ of TCA P in different plant parts of previously well-fed soybean plants. We hypothesise that stopping P supply will lead to lower δ^18^O_P_ of TCA P in source organs resulting from an increasing phosphatase activity caused by the interruption in P supply. This is tested in a single experiment in which P fluxes between plant organs are measured with ^33^P tracing while the effect of phosphatase is analysed through its effect on the δ^18^O_P_ of plant P. To our knowledge, this is the first time that such a dual isotopic approach is conducted to assess plant response to changing P nutritional status.

## Methods

### Experimental set up

Soybean (*Glycine max* cv. Toliman) was grown under controlled conditions in a glasshouse in hydroponic cultures. Soybean seeds were surface-sterilized as described by Kremer et al. [24] and germinated in sand (size 0.7–1.2 mm) saturated with ultrapure water (ddH_2_O). 8 days after seeding (DAS; stage V2, [25]), 250 plants were transferred into a hydroponic set up. 7 days later (15 DAS; stage V3), 88 soybean plants were transferred into eleven non-transparent plastics pots (size 300 mm × 200 mm × 220 mm) containing 8 L nutrient solution (eight plants per pot). From 8 to 15 DAS, the plants were supplied with a modified Hoagland nutrient solution containing 0.5 mM KH_2_PO_4_, 5 mM KNO_3_, 5 mM Ca(NO_3_)_2_, 2 mM MgSO_4_, 0.1 mM Fe chelate, 0.05 mM KCl, 0.025 mM H_3_BO_3_, 0.002 mM MnSO_4_, 0.002 mM ZnSO_4_, 0.0005 mM CuSO_4_, and 0.0005 mM Na_2_MoO_4_. The KH_2_PO_4_ used for the preparation of the nutrient solution had a δ^18^O_P_ of 12.4‰ and the water in the nutrient solution had a δ^18^O of − 10.2‰. The nutrient solution was changed regularly to reduce microbial growth and ensure optimal growth conditions for the plants. The 88 soybean plants were distributed in 11 pots representing three treatments: control, + P, and − P plants. Three replicates were used for the control, while the + P and − P treatments were replicated 4 times, with eight plants per pot.

At 15 DAS, 10 mL of a stock solution was prepared containing 1 mL of carrier-free ^33^P-PO_4_ and 9 mL of nutrient solution. The control plants received 0.6 mL of this ^33^P-containing solution per pot (equivalent to 20 MBq per pot), while the + P (sufficient amount of P supplied throughout the experiment) and − P (no P supplied for 10 days) plants received 0.9 mL per pot (equivalent to 30 MBq per pot). The plants remained in the labelled nutrient solution for 8 days (23 DAS; stage V5). After this period the plants in three pots (referred to as “control”) were harvested and the remaining eight pots were divided into two groups (four pots per group). The + P plants received the non-labelled nutrient solution with 0.5 mM KH_2_PO_4_; while the − P plants received a P-free nutrient solution. Before the transfer of the plants to the new nutrient solution, the roots were washed twice with P-free nutrient solution to avoid any carry-over of ^31^P and ^33^P into the new nutrient solution. After 10 days (33 DAS; stage V7/8) the + P and − P plants were harvested. In the middle of this period (28 DAS) 2 L P-free nutrient solution was added to each pot of the − P plants while 2 L nutrient solution containing 0.5 mM KH_2_PO_4_ were added to each pot of the + P plants.

Relative air humidity in the glasshouse ranged between 44 and 61%, with a mean of 58%. The air temperature ranged between 17 and 23 °C, with a mean of 21 °C. The plants were illuminated with 35 klux for 16 h per day. Leaf temperature of the trifoliate leaves was measured at the time of the harvest using a laser thermometer (Messtechnik Schaffhausen GmbH, Switzerland). For all treatments, the leaf temperature ranged between 19 and 22 °C, in average 20 °C, for the old leaves and between 20 and 24 °C, with an average of 22 °C for the new leaves.

### Harvest

All plants were harvested approximately at the same time of the day (ca. 11:00) and within 30 min, to reduce the effect of sampling time on the δ^18^O_P_ of TCA P, which can be up to 7‰ [26].

The plants were separated into five parts: roots, stems, old, new, and senescent leaves. Middle veins of the old and new leaves are included in the plant part “stems”, as, like the stems, they contain water with the δ^18^O of the source water [27]. The part “old leaves” includes the trifoliate leaves, which were already fully developed when the control plants were harvested. The part “new leaves” includes trifoliate leaves, which were not fully developed when the control plants were harvested, and for the + P and − P plants, also those that developed after the harvest of the control plants. The plant samples were put at − 20 °C directly after sampling. The senescent unifoliate leaves were collected and dried at 45 °C for 2 days before milling to a fine powder (< 2 mm). The unifoliate leaves were only used to determine the recovery of ^33^P taken up by the plants from the nutrient solution and the distribution of ^33^P in the plants.

### Extraction of plant material

Two different P pools were sequentially extracted from each plant part, yielding in total eight compartments. The first step of the sequential extraction with TCA is targeting mainly inorganic phosphate [28], but may also extract some labile organic P compounds (such as glucose-6-phosphate) that can be hydrolysed during the colorimetric essay [29]. For this reason, this fraction is referred to as TCA-soluble reactive P (TCA P). Two grams of frozen plant material were weighed into 60-mL plastic bottles and 40 mL of 0.3 M TCA were added. After homogenising with a Polytron^®^ (Kinematica AG, Luzern, Switzerland), the plant material was extracted for 1 h at 4 °C. The extracts were vacuum filtered using GF/F (pore size 0.7 μm; Whatman Internal Ltd.) filters before further processing for δ^18^O_P_ measurements.

The plant material remaining after the extraction with TCA was extracted with 10 M HNO_3_ following the protocol described by [30] and modified by [23]. The extraction with 10 M HNO_3_ targets stable organic P compounds, such as phospholipids and DNA, and is referred to as residual P. The material was transferred into 50-mL centrifuge tubes and 40 mL of 10 M HNO_3_ were added. The samples were put into a water bath at 50 °C and extracted overnight. On the following day, the extracts were transferred into 100-mL Erlenmeyer flasks, put on stirring plates and 0.3 M potassium permanganate (KMnO_4_) was added drop wise until a brownish precipitate formed. On the next day, 0.1 M sodium nitrite (NaNO_2_) was added until the brownish precipitate dissolved. The extracts were filtered through GF/F filters before further processing.

Additionally, total P was extracted from the senescent unifoliate leaves via ashing. 0.25 g of dried and milled plant powder was weighed into a porcelain crucible and ashed in an oven for 6 h at 550 °C. Three millilitres of 14.3 M HNO_3_ were added and the extract was diluted and filtered through a 0.2 μm filter before determining the P concentration.

The concentration of P in the 0.3 M TCA and 10 M HNO_3_ extracts and in the total P extract of the unifoliate leaves was determined colorimetrically as described by [31].

### Measurement of the ^33^P activity

The activity of ^33^P in each plant extract and in the nutrient solutions was measured with a liquid scintillation counter (TRI-CARB 2500 TR, liquid scintillation analyser, Packard Instruments, USA). Half a millilitre of each extract was mixed with 5 mL of liquid scintillation counting cocktail and measured for ten minutes. Two types of liquid scintillation counting cocktails were used, Ultima Gold for the 0.3 M TCA extracts and the nutrient solutions and Ultima Gold AB for the 10 M HNO_3_ and total P extracts (Perkin Elmer, USA). All activities were corrected for the radioactive decay and calculated back to the start of the labelling period (15 DAS).

### Purification of extracts and precipitation of silver phosphate for the δ^18^O_P_ measurements

Purification of the 0.3 M TCA extracts and precipitation of silver phosphate (Ag_3_PO_4_) followed the protocol described by [32]. One millilitre of 17.8 M H_2_SO_4_ was added during the ammonium phospho-molybdate (APM) step to facilitate the precipitation of APM. The extraction with 10 M HNO_3_ is not suitable for the determination of the δ^18^O_P_.

### Determination of δ^18^O of phosphate and water

The δ^18^O of water used for the nutrient solution was determined with the CO_2_ equilibration method [33]. For each sample, 0.2 mL were pipetted into a vacutainer, closed tightly, and flushed with a gas mixture of 0.3% CO_2_ in helium (He). After an equilibration time of 18 h at room temperature, the samples were measured with a gas bench device (Gas Bench II; Thermo Fisher Scientific Inc., Waltham, MA, USA) coupled to an isotope ratio mass spectrometer (Delta V Plus; Thermo Fisher Scientific^®^). The system was calibrated with the international standards VSMOW (Vienna Standard Mean Ocean Water; δ^18^O = 0‰), SLAP (Standard Light Antarctic Precipitation; δ^18^O = − 55.5‰ VSMOW) and GISP (Greenland Ice Sheet Precipitation; δ^18^O = − 24.8‰ VSMOW). The analytical error was lower than ± 0.06‰.

The Ag_3_PO_4_ samples were measured in triplicates by a thermal conversion elemental analyser (vario PYRO cube; Elementar, Germany) coupled to an isotope ratio mass spectrometer (IRMS; Isoprime, UK). Results were calibrated against an internal Ag_3_PO_4_ standard (Acros Organics, Geel, Belgium; δ^18^O = 14.2‰ VSMOW) and two benzoic acid standards distributed by the International Atomic Energy Agency (IAEA) in Vienna (IAEA 601: δ^18^O = 23.1‰ and IAEA 602: δ^18^O = 71.3‰ VSMOW) [34]. Each run included 82 Ag_3_PO_4_ samples, 16 samples of the Ag_3_PO_4_ standard, and eight samples of each benzoic acid standard. Analytical error calculated on replicate analyses of standards was lower than ± 0.4‰.

All oxygen isotope compositions are reported in the conventional delta notation with respect to VSMOW:

1 $$\updelta^{18} {\text{O}} = 1000*\left( {\frac{{R_{sample} }}{{R_{standard} }} - 1} \right),$$

where *R* is the ratio ^18^O/^16^O in the sample and standard, respectively.

### Calculations and statistical analysis

#### ^33^P uptake and recovery in the plants

The amount of ^33^P taken up by the plants during the experiment was calculated by subtracting the activity of ^33^P (MBq per pot) remaining in the nutrient solution before it was exchanged (23 DAS) from the activity of ^33^P (MBq per pot) in the fresh nutrient solution at the start of the labelling period (15 DAS).

The total activity of ^33^P in the plants per pot was calculated by Eq. 2:

2 $${}_{{}}^{33} P_{plant} = \mathop \sum \limits_{k = 1}^{4} \left( {TCA {}_{{}}^{33} P + residual {}_{{}}^{33} P} \right)_{k} + {}_{{}}^{33} P_{ashed}$$

where *k* are the different plant parts and *TCA* ^*33*^ *P*, *residual* ^*33*^ *P*, and ^3*3*^ *P*_*ashed*_ represents the total activity of ^33^P in the TCA P pool, residual P, and in the total P of the unifoliate leaves, respectively. The recovery of ^33^P in the plants was calculated by dividing the total activity of ^33^P in the plants per pot (Eq. 2) by the activity of ^33^P taken up by the plants during the labelling period.

#### Phosphorus derived from the labelling and non-labelling period

The amount of P taken up during the labelling period can be calculated using Eq. 3:

3 $$x = \frac{y}{{SA_{nut\;sol} }} ,$$

where *x* is the amount of P derived from the labelling period in mg per compartment and pot, *y* is the activity of ^33^P in the compartment in kBq per pot, and *SA*_*nut sol*_ is the specific activity of the P in the nutrient solution, which was taken up during the labelling period in kBq mg^−1^ P.

The amount of P derived from other sources (that is, before and after the labelling period) in mg per compartment and pot can be calculated from:

4 $$z = P_{tot} - x,$$

where *z* is the amount of P derived from before and after the labelling period in mg per compartment and pot, *P*_*tot*_ is the total amount of P in a compartment in mg per pot, and *x* is the amount of P derived from the labelling period in mg per compartment and pot.

#### Net gains and losses of phosphorus in the different compartments

The amount of P in the different compartments of the control plants was used as a baseline to calculate net gains and losses (turnover) of P in the different compartments of the − P and + P. The turnover of P derived from the labelling period in the different compartments in the − P and + P plants during the last 10 days of the experiment (23–33 DAS) were calculated using Eq. 5:

5 $$Turnover\;of\;P\;derived\;from\; the\;labelling\;period = x_{control} - x_{i} ,$$

where *x*_*control*_ is the amount of P taken up during the labelling period (mg P per compartment and pot) in the control plants and *x*_*i*_ is the amount of P taken up during the labelling period (mg P per compartment per pot) in the + P or − P plants. If the result is positive, the respective compartment is defined as sink for P derived from the labelling period (net gain), if the result is negative, the respective compartment is defined as source for P derived from the labelling period (net loss).

If a compartment was a sink or a source of P derived from the non-labelling period, was calculated using Eq. 6:

6 $$Turnover\;of\;P\;derived\;from\; the\; nonlabelling\;period = z_{control} - z_{i} ,$$

where *z*_*control*_ is the amount of P taken up during the non-labelling period (mg P per compartment and pot; 0–15 DAS) in the control plants and *z*_*i*_ is the amount of P taken up during the non-labelling period (mg P per compartment and pot; 0–15 and 23–33 DAS) in the + P or − P plants. If the result is positive, the respective compartment is defined as sink for P derived from the non-labelling period (net gain), if the result is negative, the respective compartment is defined as source for P derived from the non-labelling period (net loss).

#### Estimation of the leaf water δ^18^O

The leaf water δ^18^O was estimated based on data from a previous experiment where we looked at the daily changes of the leaf water δ^18^O and the δ^18^O_P_ of TCA P of fully developed trifoliated soybean leaves [26]. A linear regression using the relative air humidity (rH) in % in the glasshouse and the leaf temperature (*t*_leaf_) in °C as independent variable and the leaf water δ^18^O as dependent variable gave the following equation:

7 $$Leaf\;water \;\updelta^{ 1 8} {\text{O}} = 14.72 - 0.17 \cdot rH + 0.12 \cdot t_{leaf} ,$$

Adjusted R^2^ = 0.55, *p* value < 0.001.

The equilibrium between oxygen in water and in phosphate was calculated using the rearranged equation by [17]:

8 $$\updelta^{18} {\text{O}}_{\text{P}} = - 0.17 \cdot t_{leaf} + 26.2 + leaf\;water\;\updelta{}_{{}}^{18} {\text{O}}$$

### Statistical analysis

To determine the effect of each treatment, the means of the different parameters were compared by conducting a one-way analysis of variance (ANOVA) using the program R [35]. For the multiple comparisons of the means (+ P compared to − P, + P compared to control, and − P compared to control) a Tukey’s HSD test, with a significance level α of 0.05, was performed afterwards as post hoc test. To determine significant differences between the δ^18^O_P_ of TCA P in different plant parts within the same treatment a one-way ANOVA with a subsequent Tukey’s HSD (α = 0.05) was performed as post hoc test. Pooled standard deviations were calculated for *M*_d_, total amount of P per compartment, P in each compartment derived from the labelling or non-labelling period, and the δ^18^O_P_ of TCA P.

## Results

### Uptake of water, ^31^P, and ^33^P during the experiment

The total water uptake per pot during the experiment (15–23 DAS in case of the control and 15–33 DAS in case of the + P and − P plants) was 1 L for the control and 4 L for the + P and − P plants.

The net uptake of P during the experiment (15–23 DAS in case of the control; 15–33 DAS in case of the + P and − P plants) was 46 mg, 145 mg, and 43 mg as average per pot for the control, + P, and − P plants, respectively. The average uptake of ^33^P per pot was 8.5 MBq per pot, 11.4 MBq per pot, and 12.1 MBq per pot for the control, + P, and − P plants, respectively. 42, 39, and 41% of ^33^P added in the nutrient solution was taken up by the control, + P, and − P plants. A small amount of ^33^P was found in the nutrient solution of the + P and − P plants at 33 DAS (Table 1).

**Table 1:** Phosphorus content (mg P) and activity (MBq) in the nutrient solution at the beginning of the labelling period [8 L; 15 days after seeding (DAS)], remaining at the end of the labelling period (7 L; 23 DAS), in the new nutrient solution after the labelling period (8 L; 23 DAS), and at the end of the experiment (7 L; 33 DAS)

	Start (15 DAS)		End of labelling (23 DAS)		New nutrient solution (23 DAS)		End of experiment (33 DAS)	
	^33^P (MBq)	^31^P (mg P)	^33^P (MBq)	^31^P (mg P)	^33^P (MBq)	^31^P (mg P)	^33^P (MBq)	^31^P (mg P)
Control	20 (0.3)	108 (3)	11.5 (1.6)	62 (7)	na	na	na	na
+ P	29.4 (1.3)	104 (4)	18.0 (1.7)	51 (6)	0	104^a^ (3)	0.17 (0.08)	43 (21)
− P	29.5 (0.7)	104 (3)	17.5 (1.0)	54 (2)	0	0^a^	0.04 (0.006)	7 (0.7)

Mean values with n = 3 for the control and n = 4 for the + P and − P. Values in brackets are the standard deviation

*na* not applicable

^a^After 5 days (at 28 DAS) 2 L nutrient solution was added to the pots of the + P treatment and 2 L P-free nutrient solution was added to the pots of the − P treatment

### Biomass and phosphorus concentrations in the plants

The average dry mass (*M*_d_) of the plants per pot increased significantly from the control (22 g *M*_d_ per pot) to the + P and − P plants (39 and 41 g *M*_d_ per pot, respectively) (ANOVA; *p* value < 0.02). The difference in *M*_d_ per pot between + P and − P plants was not significant. The *M*_d_ of the old leaves did not change significantly between the control and the + P and − P plants (Table 2).

**Table 2:** Summary of the average dry mass (*M*_d_) for the different compartments and treatments

	Dry mass (g per pot)			
	Roots	Stems	Old leaves	New leaves
Treatment				
Control	8.1a	6.5a	4.7ab	2.5a
+ P	16.7b	11.0b	4.8a	6.3b
− P	14.3ab	13.3b	4.3b	8.7b
Pooled SD	4.1	1.4	0.3	1.4
Source of variation (*p* value)				
Treatment	0.07	< 0.001	0.08	0.001

Values are means with n = 3 for the control and n = 4 for the + P and − P. Letters behind values indicate significant differences between the different treatment within the same plant parts based on a Tukey’s HSD test with α = 0.05. Pooled standard deviation (pooled SD)

The total amount of P in the control, + P, and − P plants was 67.2, 153.2, and 58.3 mg respectively. Although the total amount of P in the + P plants was about three times higher compared to the − P plants, we did not observe any visible signs of P limitation in case of the − P plants. The amount of P in the different compartments is shown in Table 3.

**Table 3:** Amount of P in the trichloroacetic acid-soluble reactive P (TCA P) and in the residual P pool derived from the labelling period [15–23 days after seeding (DAS)], from the non-labelling period, and the total amount of TCA P in each compartment

	Labelling period				Non-labelling period				Sum (0-33 DAS)			
	mg P per compartment											
	Roots	Stems	Old leaves	New leaves	Roots	Stems	Old leaves	New leaves	Roots	Stems	Old leaves	New leaves
TCA P												
Control	17.6a	6.7a	12.5a	3.0a	6.9a	4.9a	5.8a	0.9a	24.5a	11.6a	18.3a	3.9a
+ P	10.9a	12.0a	9.3ab	6.3b	36.8b	26.9b	9.9a	17.2b	47.7b	38.9b	19.1a	23.5b
− P	9.8a	9.5a	3.0b	5.2ab	4.0a	4.5a	0.7b	2.2a	13.8c	14.0a	3.7b	7.4a
Pooled SD	4.6	3.3	3.7	1.4	10.6	11.1	4.3	13.5	3.9	4.2	4.0	4.2
Source of variation (*p* value)												
Treatment	ns	ns	0.03	0.05	< 0.001	< 0.001	< 0.001	< 0.001	< 0.001	< 0.001	0.001	< 0.001
Residual P												
Control	1.6a	0.9a	1.7a	1.0a	1.1a	0.9a	1.2a	0.5a	2.6a	1.8a	2.9a	1.5a
+ P	1.5a	2.5b	1.4a	2.5a	3.8b	5.1b	2.8a	4.4b	5.3b	7.7b	4.2a	6.8b
− P	2.4a	4.7c	1.5a	6.9b	1.2a	0.6a	0.5a	1.6ab	3.6a	5.2b	2.0a	8.5b
Pooled SD	0.6	0.3	0.2	0.5	0.9	0.9	1.9	3.0	1.4	1.0	1.4	2.1
Source of variation (*p* value)												
Treatment	ns	< 0.001	ns	< 0.001	0.006	< 0.001	ns	0.05	0.1	< 0.001	ns	0.006

In the case of the control, the non-labelling period includes the first 15 days (0–15 DAS). In the case of the + P and − P, the non-labelling period represents the time from 0 to 15 DAS and from 23 to 33 DAS. Letters behind values indicate significant differences between the treatments within the same plant part based on a Tukey’s HSD test with α = 0.05

**Fig. 1:**
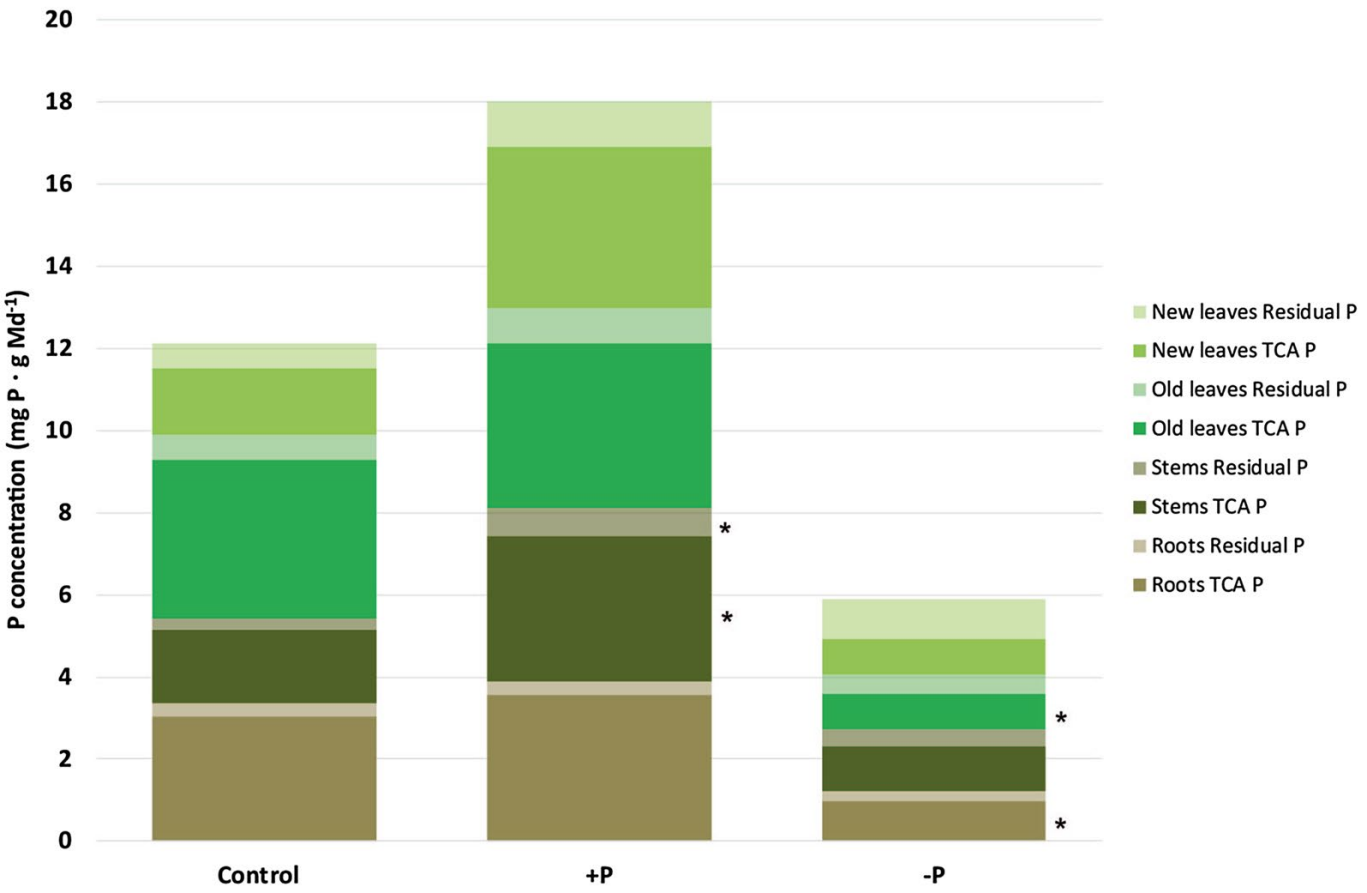
Phosphorus concentration (mg P per g dry mass) in the two different P pools [trichloroacetic acid soluble-reactive P (TCA P) and residual P] and four different plant parts for the control, + P, and − P plants. Error bars are ± SD with n = 3 for the control and n = 4 for the + P and − P. Significant differences between treatments within the same plant part are indicated by asterisks; Tukey’s HSD test with α = 0.05

We observed significant differences in the concentration of TCA P for all four parts between the + P and − P plants. The difference in the concentration of TCA P between the control and − P plants was also significant in the case of the roots and the old leaves (Fig. 1). Significant differences in the concentration of residual P were only observed in case of the stems.

### Distribution of ^33^P in the different compartments

**Fig. 2:**
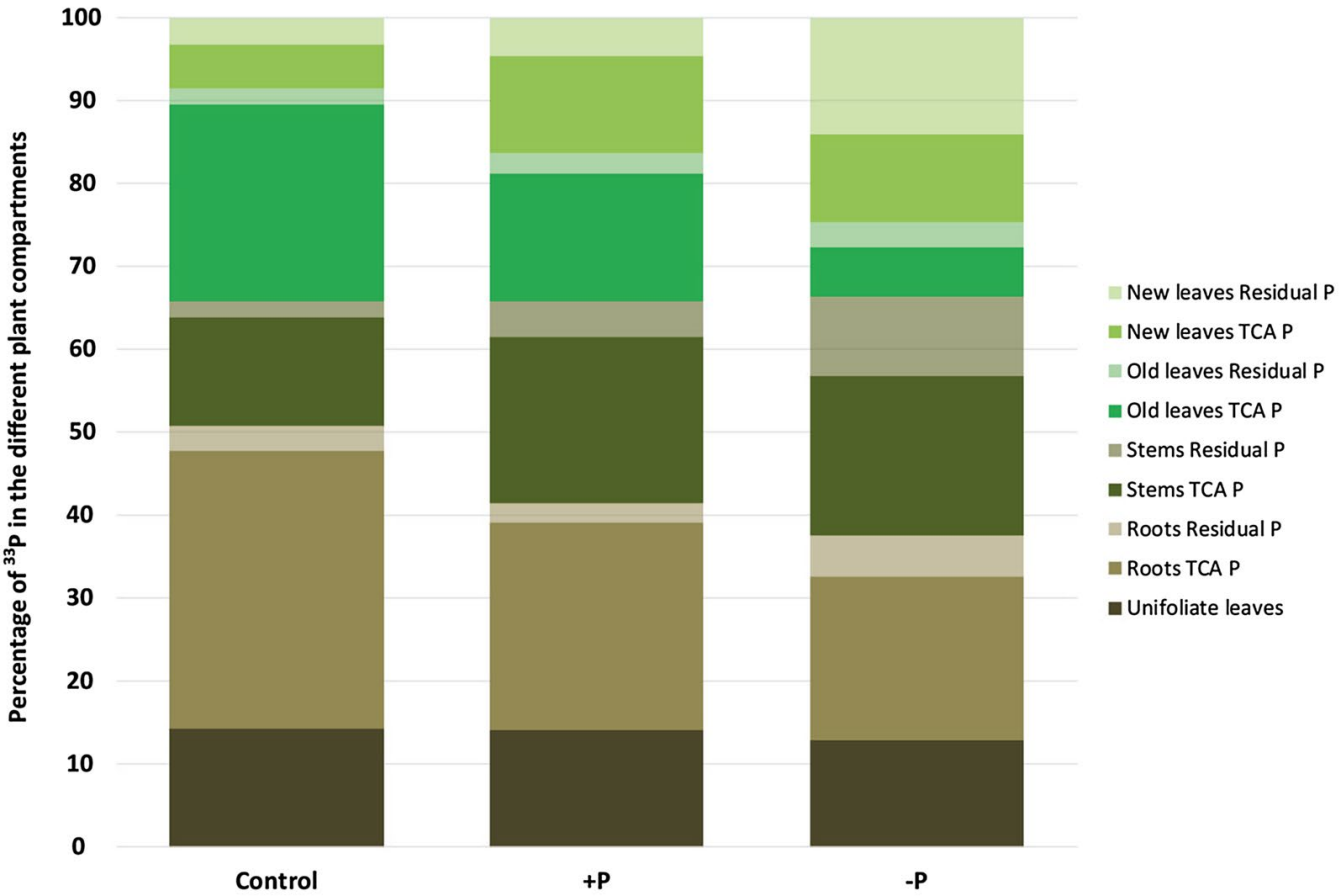
Proportion of ^33^P in the different plant compartments for the control, + P, and − P. 100% is equivalent to 9.5 MBq, 12.4 MBq, and 11.9 MBq in the case of the control, + P, and − P plants, respectively

The total activity of ^33^P found in the plants per pot was 9.5, 12.4, and 11.9 MBq for the control, + P, and − P plants, respectively. Thus, around 100% of the ^33^P, which was taken up, was recovered in the plants. The proportional distribution of the total activity of ^33^P among the different compartments is shown in Fig. 2. The proportion of ^33^P in the TCA P was higher than in the residual P (Fig. 2). The highest proportion of ^33^P was found in the TCA P of the roots and old leaves in the case of the control and in the roots and stems in the case of the + P and − P plants (Fig. 2). The proportion of ^33^P in the residual P increased significantly from the control and + P plants to the − P plants (ANOVA, *p* value < 0.001; Fig. 2). Around 14% of the ^33^P was found in the total P of the unifoliate leaves (UL) in case of all three treatments.

### Amount of phosphorus derived from the labelling period and the non-labelling period

Using Eqs. 3 and 4, the amounts of P derived from the labelling period and non-labelling period in mg P per compartment were calculated (Table 3).

**Fig. 3:**
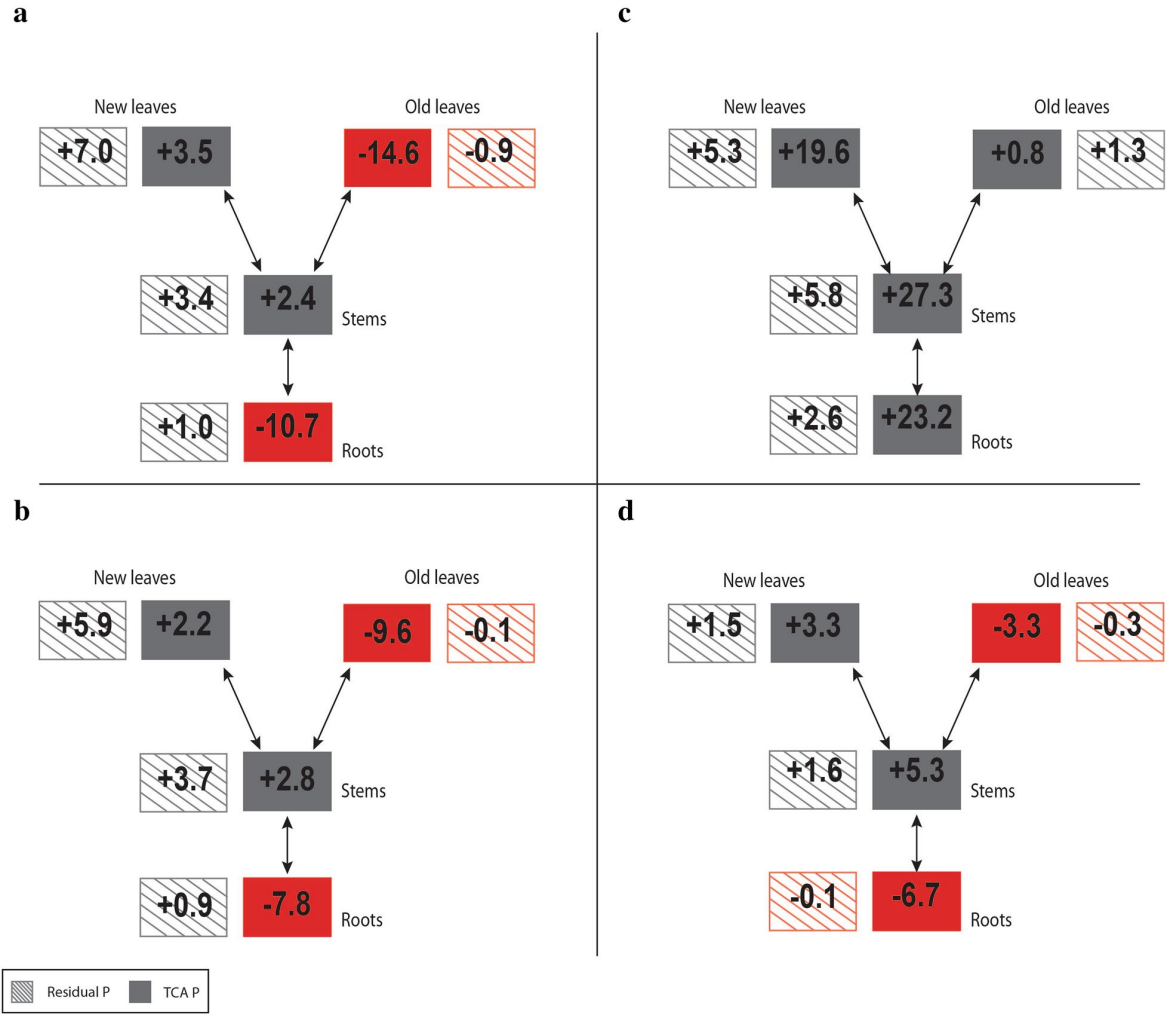
Net gains and losses of P in mg P in the different compartments of the − P (**a**, **b**) and + P (**c**, **d**) plants, for the labelling period (15–23 DAS; **b**, **d**) and the days after the labelling period (23–33 DAS; **a**, **c**). For clarity reasons, standard deviations are not shown. Red indicates net P losses; grey indicates net P gains

Net gains and losses of P in the different compartments of the − P and + P plants for the period 23–33 DAS were calculated using Eqs. 5 and 6 and are shown in Fig. 3. The main sinks in the − P plants for the P derived from the labelling period was the residual P of the roots, stems, and new leaves and to a lesser degree also the TCA P pool of the stems and new leaves (Fig. 3). The main sources of P in the − P plants were the TCA and residual P in the old leaves and the roots from the labelling and non-labelling periods (Fig. 3). In the case of the + P plants, the higher proportion of P was derived from the period referred to as “non-labelling period”. The main sink for the P derived from the labelling period in the + P plants was the residual and TCA P of the new leaves and of the stems (Fig. 3).

### δ^18^O_P_ of TCA P, leaf water δ^18^O and equilibrium

The δ^18^O_P_ of TCA P extracted from the old and new leaves in all three treatments was more positive than the phosphate source (12.4‰; Table 4), whereas in the roots and stems it was like that of the phosphate source (Table 4). Significant differences were observed between the δ^18^O_P_ of TCA P in the old leaves of the control, + P and − P plants and in the new leaves between the control and − P plants and between the + P and − P plants.

**Table 4:** δ^18^O_P_ of trichloroacetic acid-soluble reactive P (TCA P) of the four different parts (roots, stems, old and new leaves) of the plants for the three different treatments (control, + P and − P)

	δ^18^O_P_ (‰)			
	Roots	Stems	Old leaves	New leaves
Treatments				
Control	12.0a, A	14.9a, B	29.8a, C	25.9a, D
+ P	11.5a, A	13.5a, B	27.4b, C	25.7a, D
− P	12.0a, A	13.9a, B	23.8c, C	24.2b, C
Pooled SD	0.7	1.1	0.9	0.7
Source of variation (*p* value)				
Treatment	ns	ns	< 0.001	0.02

Values are means with n = 3 for the control and n = 4 for the + P and − P plants. Small letters indicate significant differences between treatments within the same plant part; capital letters indicate significant differences between plant parts within the same treatment (Tukey’s HSD; α = 0.05). Pooled standard deviation (pooled SD)

*ns* not significant

Based on Eq. 7 the estimated leaf water δ^18^O at a relative air humidity of 58% is between 7.3 and 7.7‰ in case of the old leaves (leaf temperature range 19–22 °C) and between 7.4 and 7.9‰ in case of the new leaves (leaf temperature 20–24 °C). Using these values and the measured leaf temperatures, equilibrium calculated with Eq. 8 is between 29.7 and 30.6‰ for the old leaves and between 29.6 and 30.7‰ for the new leaves. The water δ^18^O in roots and stems is usually close to the source water δ^18^O (− 10.2‰ in this study). Thus, using a temperature range of 18–22 °C, the equilibrium for stems and roots is between 12.3 and 12.9‰.

## Discussion

Omitting P in the nutrient solution did not affect the dry mass of our soybean plants and − P plants did not show visible signs of P limitation like darker leaves or stunted growth. This is most likely due to the high (0.5 mM P) amount of P supplied in the nutrient solution and the short duration of the P omission (10 days). Indeed, Fredeen et al. [36] observed visible signs of P limitation in soybeans grown in low P (0.01 mM P) nutrient solution from the beginning of the experiment only after 10 days. Nevertheless, we observed changes in the distribution of P in the different plant parts and P pools, which indicates a change in plant metabolism.

### Plant water δ^18^O and the δ^18^O_P_ of TCA P

The δ^18^O_P_ of TCA P of the old leaves of the control plants and of the stems and roots in general were relatively close to the calculated equilibrium with the plant water [Table 3; 29.7–30.6‰ (equilibrium range old leaves), 12.3–12.9‰ (equilibrium range stems and roots)]. Differences between calculated equilibrium and measured δ^18^O_P_ of TCA P values are not caused by the leaf water δ^18^O, even though the equilibrium values are calculated using several assumptions. The + P and − P plants were harvested within half an hour and thus the leaf water δ^18^O should not vary greatly [26]. Furthermore, the variability of the δ^18^O_P_ of TCA P among the four replicates per treatment was low, indicating that the sampling time (< 30 min) did not affect the δ^18^O_P_ of TCA P. In addition, we consider that transpiration, the main driver behind changes in the leaf water δ^18^O, of the + P and − P plants was the same, as + P and − P plants did not differ in their water uptake and biomass. Based on Eq. 8 the difference in leaf water δ^18^O between old and new leaves would be rather small and we thus assume that leaf water δ^18^O values are not the cause for lower δ^18^O_P_ of TCA P values of the new leaves compared to the old leaves.

### Changes in the δ^18^O_P_ of TCA P and the distribution of ^33^P

The amount of P and distribution of ^33^P within the plants indicate that P was mobilised and translocated between different plant parts in the − P and + P soybean plants in our study (Table 3 and Fig. 3). This is not surprising as P is continuously circulated between plant parts even when plants are not P limited as shown by [37]. In line with this, the translocated P, derived from the labelling period was mainly found in the TCA P of the new leaves and stems (Fig. 3). In case of the − P plants, the omission of P resulted in an enhanced re-mobilisation of P from residual P in the old leaves and translocation of P from the TCA P in the old leaves and roots to the new leaves and stems (TCA and residual P; Fig. 3). Mobilisation and translocation of P is a common response of plants to the omission of P and was observed for a wide range of plant species (for example soybean, mashbean and mungbean [38], citrus trees [39], beans [40], *Brachiaria* [41], and peas [42]). Except for the residual P in the old leaves, P derived from the labelling period, was translocated and used for the synthesis of residual P in the roots, stems, and new leaves. There was a small efflux of P, which had been taken up during the labelling period, to the nutrient solution in the last 10 days of the experiment for both + P and − P plants (Table 1). An increased efflux of P has been observed for plants grown at high P levels [43]. This could help the plant to maintain P homeostasis and possibly explain the efflux in case of the + P plants. The efflux of ^33^P could also have been caused by senescent root tissue entering the nutrient solution, which was most likely the case for the − P plants.

Re-mobilisation of residual P, i.e. organic P, as observed for example the old leaves of the − P plants, involves hydrolysing enzymes such as acid phosphatase [44–46]. A discrimination factor of − 10‰ was reported for acid phosphatase, leading to a depletion in ^18^O of the released phosphate [22]. To estimate the δ^18^O of phosphate released by hydrolysing enzymes, we would need to know the δ^18^O of residual P (organic compounds) inside the plants. At present, we can only approximate this value by considering that organic compounds in a cell have the same δ^18^O_P_ as phosphate inside the cell [47]. If we assume a δ^18^O_P_ of residual P of 27.4‰ (δ^18^O_P_ of TCA P in the old leaves of the + P plants) and a leaf water δ^18^O values between 7.2 and 7.5‰, phosphate released due the hydrolysis of residual P by acid phosphatase would have a δ^18^O_P_ value around 20‰. The release of such light phosphate could explain our observed differences in the δ^18^O_P_ of TCA P of + P and − P plants as shown with the following calculations and considerations.

We observed a net P loss of 0.9 mg P from residual P in the old leaves of the − P plants during the last 10 days of the experiment (Fig. 3). The total amount of TCA P in the old leaves was 3.7 mg (Table 3). Thus, P mobilised from residual P in the old leaves could potentially make up 25% of the TCA P in the old leaves of the − P plants. Using a simple mass balance, the δ^18^O_P_ of TCA P in the old leaves of the − P plants would be around 25.5‰. Such a mixing could also explain the lower δ^18^O_P_ of TCA P in the new leaves of the − P plants compared to the + P plants. Light phosphate produced by phosphatases in developed leaves could be transported to developing leaves, diluting the TCA P pool and leading to lower δ^18^O_P_ values.

As phosphate is transported from the old leaves to the new leaves via the stems, such a mixing could also explain the slightly, but not significantly, lower δ^18^O_P_ of TCA P values of the stems in case of the + P and − P plants compared to the control plants. Doing a similar calculation for the stems of the − P plants as for the old leaves of the − P plants, 18% of the TCA P in the stems would be P originating from the hydrolysis of residual P based on δ^18^O_P_ values and by 17% based on Fig. 3 and Table 3.

The stems of the + P plants present a more complicated case, since P continues to be taken up from the nutrient solution. Phosphorus in the stems increased by 70% (Fig. 3 and Table 3). Using the δ^18^O_P_ of TCA P, only 7% would originated from P released by hydrolysis, whereas 63% would be P taken up from the nutrient solution. 7% of the amount of TCA P in the stems is 2.7 mg and are very close to the amount of residual P mobilised from the old leaves (3.3 mg). It is possible that changes of the δ^18^O_P_ of TCA P in the stems of the + P and − P plants compared to the control plants reflect a mixing of different phosphate sources.

Translocation of P from the TCA P was also observed in case of the roots (+ P and − P plants; Fig. 3), but contrary to the δ^18^O_P_ of TCA P in the old leaves, the δ^18^O_P_ of TCA P in the roots did not change from the + P to the − P plants. Unlike in the old leaves, no net loss of P from the residual P in the roots during the last 10 days of the experiment was observed. Thus, contribution of phosphate released from residual P by hydrolysing enzymes to the TCA P pool in the roots was negligible. In the case of the + P plants also the high P influx from the nutrient solution and the high amount of TCA P in the roots might have masked any contribution of P with a lighter δ^18^O_P_ value.

The δ^18^O_P_ of TCA P in the roots, as well as in the stems of the − P plants was close to the calculated equilibrium as well as to the δ^18^O_P_ of the P source in the nutrient solution (12.4‰). However, the − P plants did not receive any P for the last 10 days of the experiment and most of the TCA P in the roots was translocated to other compartments (Fig. 3). This strongly suggests that in the roots and stems, the δ^18^O_P_ signature of the P source is reset by O exchange with water mediated by inorganic PPase and other acid anhydride hydrolases [18, 20]. Acid anhydride hydrolases are also present in stems and roots [48], as they are important for loading and unloading of the xylem and phloem in the roots and stem [49–52].

## Conclusions

By using a dual isotopic approach (^33^P and the δ^18^O_P_) we simultaneously investigated P translocation and the enzymatic release of P due to a change in P nutrition. We showed that stopping P supply for 10 days lead to a translocation of P from source organs, mainly from residual P, to sink organs of up to 25%. Furthermore, the δ^18^O_P_ of TCA P in source organs was significantly lower compared to sink organs due to the enzymatic hydrolysis of organic P. Through further studies with different plant species, the δ^18^O_P_ can become a useful tool to investigate early responses of plants to P limitation—in field and laboratory studies—before any visible signs of P limitation are observed. This requires also that more fractionation factors associated with enzymes involved in P mobilisation and translocation in plants are characterised, as well as a method to determine the δ^18^O_P_ of organic P in plants.

## Abbreviations

ANOVA: analysis of variance
APM: ammonium phospho-molybdate
DAS: days after seeding
ddH_2_O: ultra-pure water
δ^18^O: isotopic composition of oxygen
δ^18^O_P_: isotopic composition of oxygen associated to phosphorus
GB: gas bench
GISP: Greenland ice sheet precipitation
He: helium
IAEA: International Atomic Energy Agency
IRMS: isotope ratio mass spectrometer
*M*_d_: dry mass
O: oxygen
P: phosphorus
− P: no P supplied for 10 days
+ P: sufficient amount of P supplied throughout the experiment
PPase: pyrophosphatase
^33^P-PO_4_: ^33^P labelled phosphate
P_tot_: total P
R: isotopic ratio of ^18^O to ^16^O
Residual P: P extracted by 10 M HNO_3_ after extraction with TCA
Residual ^33^P: activity of ^33^P in the residual P
rH: relative humidity
SA: specific activity
SA_nut sol_: specific activity in the nutrient solution
SLAP: standard light Antarctic precipitation
TCA: trichloroacetic acid
TCA P: trichloroacetic acid soluble reactive phosphorus
TCA ^33^P: activity of ^33^P in TCA P
TC/EA: thermal conversion elemental analyser
T_leaf_: leaf temperature
UL: unifoliate leaves
VSMOW: Vienna standard mean ocean water
x: amount of P derived from the labelling period
x_control_: amount of P taken up during the labelling period in the control plants
x_i_: amount of P taken up during the labelling period in the + P or − P plants
y: activity of ^33^P in a certain compartment
z: amount of P derived from before and after the labelling period
z_control_: amount of P derived from before the labelling period in the control plants
z_i_: amount of P derived from before and after labelling period in the + P or − P plants
